# Chronic Exposure to Polystyrene Microplastic Fragments Has No Effect on Honey Bee Survival, but Reduces Feeding Rate and Body Weight

**DOI:** 10.3390/toxics11020100

**Published:** 2023-01-21

**Authors:** Yahya Al Naggar, Christie M. Sayes, Clancy Collom, Taiwo Ayorinde, Suzhen Qi, Hesham R. El-Seedi, Robert J. Paxton, Kai Wang

**Affiliations:** 1 General Zoology, Institute for Biology, Martin Luther University Halle-Wittenberg, Hoher Weg 8, 06120 Halle (Saale), Germany; 2 Zoology Department, Faculty of Science, Tanta University, Tanta 31527, Egypt; 3 Department of Environmental Science, Baylor University, Waco, TX 76706, USA; 4 Institute of Apicultural Research, Chinese Academy of Agricultural Sciences, Beijing 100093, China; 5 Pharmacognosy Group, Department of Pharmaceutical Biosciences, Biomedical Centre, Uppsala University, P.O. Box 591, SE-751 24 Uppsala, Sweden; 6 Department of Chemistry, Faculty of Science, Menoufia University, Shebin El-Koom 32512, Egypt

**Keywords:** plastic pollution, honey bees, pollinators, polystyrene, chronic exposure

## Abstract

Microplastics (MPs), in the form of fragments and fibers, were recently found in honey samples collected in Ecuador as well as in honey bees collected from Denmark and China. However, little is known about how MPs impact bee health. To fill this knowledge gap, we investigated the potential toxicity of irregularly shaped polystyrene (PS)-MP fragments on honey bee health. In the first experiment of its kind with honey bees, we chronically exposed bees with a well-established gut microbiome to small (27 ± 17 µm) or large (93 ± 25 µm) PS-MP fragments at varying concentrations (1, 10, 100 µg mL^−1^) for 14 days. Bee mortality, food consumption, and body weight were all studied. We found that chronic exposure to PS-MP fragments has no effect on honey bee survival, but reduced the feeding rate and body weight, particularly at 10 µg PS-MP fragments per mL, which may have long-term consequences for honey bee health. The findings of this study could assist in the risk assessment of MPs on pollinator health.

## 1. Introduction

In the past ten years, attention to the emerging pollutant of microplastics (MPs) has increased [1,2,3], and they are now known to be present in a wide range of environmental matrices, including the air, soil, and water [4,5,6]. There is a pressing need to understand the effects of MPs on exposed organisms.

MPs are classified as polymeric particles with a plasticizer component; by definition, they are no larger than 5 mm in diameter [7,8]. They are further divided into two subgroups, namely, primary and secondary MPs. The majority of those in the primary subgroup are made directly as microscopic materials and are frequently used in consumer goods such as cosmetics, detergents, and cleaning products. Secondary MPs are created when larger plastic materials deteriorate (break down) as a result of natural weathering processes in the air or water. For instance, municipal sewage sludge, road wear particles, and tires have all been identified as potential major sources of microplastics in the environment [9,10]. Given the vast quantity of plastics entering the environment, most MPs in the environment are thought to be secondary MPs [11,12].

Pollinators are inextricably linked to the natural environment and modern food production; they maintain a healthy, genetically diverse plant ecosystem, are essential for crop pollination, and are thus critical to food security for many human populations around the world [13,14,15,16]. Honey bees actively interact with plants, air, soil, and water near the hive and, as a result, pollutants from these sources are transferred into honey bees and hive products [17]. Indeed, honey bees come into contact with every environmental compartment while collecting nectar, honeydew, pollen, and other plant exudates. If MPs pollute the nearby compartments, they will eventually be acquired by honey bees and enter hive products [18]. 

Microplastics were recently found in 12% of the honey samples collected in Ecuador, consisting primarily of polyethylene, polypropylene, and polyacrylamide polymers [19], as well as in honey bees collected from Denmark and China in the form of fragments (52%) and fibers (38%) [20]. However, little is known about how MPs impact bee health [18], and those results that have been reported are inconsistent. For example, some research suggests that honey bees may be more vulnerable to viral infections when they consume spherical polystyrene microplastics (PS-MPs) [21]. They may also have less bacterial diversity in their guts, and exhibit changes in gene expression linked to oxidative damage, detoxification, and immunity [22]. Surprisingly, polystyrene plastic at the nano size (100 nm) impacted honey bee health more than PS at the micro-size [23]; after honey bees ingested nano-PS (10^5^ particles/mL), an accumulation of pollen in the rectum was found, possibly preventing digestive enzymes from contacting pollen and interfering with the utilization of nutrients in pollen by the gut microbiota, resulting in energy deficiency and significant weight loss. Furthermore, nano-PS increased the multiplication of *Hafnia alvei* in host guts [23] and may promote the spread of pathogenic bacteria from guts to hemolymph, resulting in increased honey bee mortality. PS microfibers, on the other hand, did not kill honey bees in acute toxicity tests [24]. In addition, the health and cognition of honey bees are minimally impacted by acute and chronic consumption of polyethylene (PE) MPs [25]. Studies have yet to investigate the potential effects of irregularly shaped MP fragments on honey bee health. This is especially relevant as MP fragments may be more toxic than spheres in other organisms, including insects [26,27,28].

We therefore designed a study to investigate the potential toxicity of PS-MP fragments on honey bee health, the first time in which honey bees have been exposed to PS-MPs in a controlled laboratory setting. To achieve this, we chronically exposed bees with a well-established gut microbiome to small or large PS-MP fragments at varying concentrations for 14 days. Bee mortality, food consumption, and body weight were all recorded. We hypothesized that exposing bees to PS-MP fragments would block their alimentary canal and affect their feeding rates and body weight.

## 2. Materials and Methods

### 2.1. MP Preparation 

**Figure 1:**
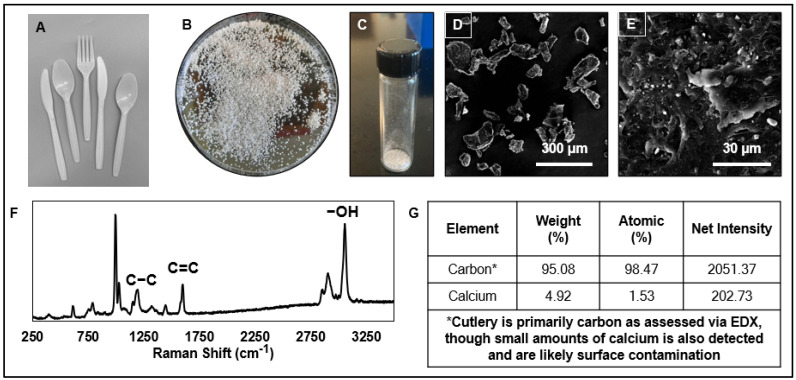
Physicochemical characterization of the microplastic particles used in this study. (**A**) Photograph of the white plastic cutlery prior to grinding. (**B**) Photograph of the plastic after grinding. (**C**) Photograph of size-separated microplastic particles. The mean (±SD) size of the first particle size population is 93 ± 23 μm. The mean (±SD) size of the second particle size population is 27 ± 17 μm. (**D**,**E**) Scanning electron micrographs of the microplastic particle size population with scale bars of 300 μm and 30 µm, respectively. (**F**) Raman spectroscopy of particles indicating polystyrene polymeric structure. (**G**) Elemental composition, as measured by energy-dispersive X-ray (EDX) spectroscopy, indicates the presence of carbon and calcium. Both particle size populations exhibited the same Raman and EDX spectral patterns.

Plastic cutlery was purchased from a local grocery store (Walmart, Waco, TX, USA) in store-brand cardboard packaging. In the laboratory, the items were carefully removed after a 70% ethanol spray had been applied to the package opening. The plastic items were placed in a blender for grinding. The resultant microplastics were transferred to sieves where particles greater than 500 μm were removed and the remaining particles were separated into 1–50 μm PS-MPs (labeled as the first particle size population) and 50–100 μm PS-MPs (labeled as the second particle size population). The particle populations were stored in glass scintillation vials and characterized for physicochemical characteristics. Photographs describing the steps of this procedure are provided in Figure 1.

### 2.2. Physical Characterization of MPs

Particles were characterized for the size and size distribution. Using scanning electron microscopy, over 50 images were collected of each particle population (Focused Ion Beam Scanning Electron Microscope (FIB-SEM), Versa 3D, FEI Company (Hillsboro, OR, USA). From the images, individual particles were measured (Wacom Cintiq 22HD Workstation, Olympus CellSens, Houston, TX, USA). The average size was 27 ± 17 μm (mean ± SE) for the first population (small size), while the average size for the second population (large size) was 93 ± 25 μm (Figure 1).

### 2.3. Chemical Characterization of MPs

The chemical composition of the MP populations was assessed via Raman spectroscopy (ultra-high-vacuum (UHV) tip-enhanced Raman apparatus, Thermo Fisher Scientific, Denver, CO, USA) and energy-dispersive X-ray spectroscopy (EDS by EDAX, AMETEK, Inc., Berwyn, PA, USA). From the Raman spectra, the pattern indicated the presence of polystyrene polymer due to its characteristic signatures at the at 1630 to 1000 cm^−1^ reference band, representing the styrene vinyl bond (specifically 1155 for C-C stretch and 1583 for C=C stretch). The Raman pattern for the second microplastic particle population was identical to the pattern for the first. Similarly, the peaks indicating the presence and intensities of individual elements (from highest intensity to lowest: carbon and calcium) were the same for both particle populations (Figure 1).

### 2.4. Experimental Setup

#### 2.4.1. Honey Bees

Three colonies of local *Apis mellifera* maintained in the General Zoology apiary at Martin Luther University Halle-Wittenberg, Germany, were used from July to August 2022. Prior to any research activities, the colonies were inspected visually for *Varroa destructor* mites; none were found (mite infection was below our detection threshold). We also screened the colonies for six common honey bee viral targets (DWV-A, DWV-B, BQCV, SBV, CBPV, SBPV; see [29] for details) by qPCR; all three colonies were virus-free (below the detection threshold of C_q_ = 40, i.e., no qPCR signal).

#### 2.4.2. Microbiome Feeding and Exposure Protocol

**Figure 2:**
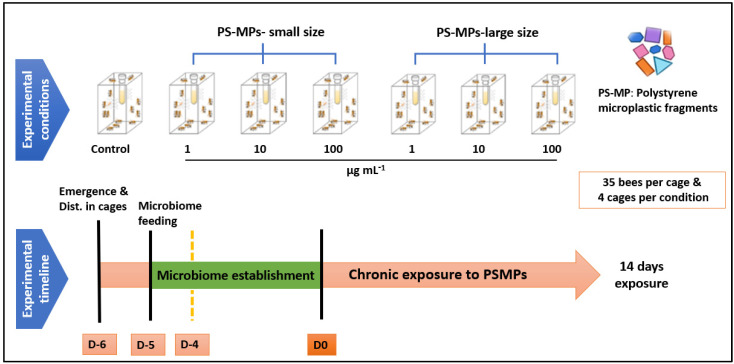
Experimental design. Upper row: treatments (four cages per treatment); lower row: timing of microbiome establishment (D-5), PS-MPs treatment (D0).

The gut microbial community of the western honey bee (*Apis mellifera*) is relatively stable, and it is presumed to improve host health and defend against parasites and pathogens [30,31,32,33]. In addition, it has been recently shown to protect honey bees from the risks of polystyrene microplastics exposure [22]. As a result, it is critical to expose bees to PS-MP fragments after the gut microbiome has been established. To establish the microbiome in the experimental bees, the brood frames from the three honey bee colonies were transferred into an incubator (35 ± 1 °C in darkness, 60% relative humidity (RH)) overnight and newly emerged bees < 24 h old were collected for use in the experiments. Then, ten bees from in-hive (nurse) bees from the source colonies were collected, brought into the laboratory, anesthetized on ice, and then sacrificed by dissecting their gastrointestinal tracts (gut sections: midgut/ventriculus, ileum, and rectum, excluding the crop) under sterile conditions to prepare the gut homogenate. The dissected gastrointestinal tracts were then pooled, homogenized in 3 mL sterile 1:1 sucrose-water with a sterile pestle, and finally diluted 3:8 with sterile 1:1 sucrose–water solution to obtain the gut homogenate (initial gut microbiota). This mixture was then given to our freshly emerged, experimental bees housed in metal cages (10 × 10 × 6 cm, *n* = 35 bees per cage) via bulk feeding, i.e., providing them with a feeding tube containing the mixture for 24 h. Then the tube was removed and a new one containing 1:1 sucrose–water solution was provided for 4 days to allow the microbiome to establish. These methods follow [34] (Figure 2). This approach of establishing the gut microbiota was chosen because it produces bees with gut communities that are comparable to those found in regular bee colonies [35,36,37]. 

The cages were placed into incubators at 30 ± 1 °C and 50% RH. In total, we had seven treatment groups: control, 1, 10, 100 µg mL^−1^ of PS-MPs fragments of small size, 1, 10, and 100 µg mL^−1^ of PS-MPs fragments of large size, with four cages per treatment and 35 bees per cage. The bees were exposed chronically for 14 days to PS-MPs, as illustrated in Figure 2. Stock concentrations of PS-MP particles were prepared at 1000 µg mL^−1^ and stored at 4 °C in the dark. To disaggregate the particles, parent stock solutions were sonicated using a SONICA-2200 E (20 kHz; 750 W) sonicator prior to each use. Then, test solutions of both small and large PS-MPs were prepared freshly at 1, 10, and 100 µg mL^−1^ using 50% (*w*/*w*) sucrose solution before use. The various sizes and concentrations of MPs were chosen to represent a range of observed titers in the honey and honey bees [19,20] and to be comparable to those tested in previous research [21,22,24].

Before the bees were exposed to PS-MPs, we starved them for two hours to induce them to consume sugar syrup containing PS-MP fragments. Both honey bee mortality and the volume of sugar syrup consumed from 2 mL tube feeders was recorded daily. Following a daily survival check, the feeder weights were recorded and then refilled with PS-MP dilutions kept at 4 °C, and the weights were once again recorded. Daily calculations were then made based on the feeders’ weight differences. The feeding rate was calculated by dividing the calculated amount of sugar syrup consumed each day by the number of animals, presuming that the mass of food consumed was uniform for each bee. 

At the end of the experiment, we collected subsamples (six bees per cage) (i.e., 24 bees per treatment) to check for potential adverse effects of PS-MPs fragments on the body weight of the bees. To do so, the bees were dried in an incubator at 50 °C for 24 h to achieve a constant weight, and the dry weights were determined using an analytical balance (accuracy ± 0.1 mg).

### 2.5. Statistical Analysis

All statistical analyses and data visualizations were performed in GraphPad Prism 8.0 (GraphPad, La Jolla, CA, USA). The normality of the data was assessed by the use of the Kolmogorov–Smirnov test and homogeneity of variance was determined with Levene’s test. The body weight data were log_10_-transformed to better approximate normality and homogeneity of variances. Due to technical issues, one cage from both the control and bees exposed to 100 µg mL^−1^ of large-sized PS-MP fragments were excluded from the experimental bees. Right-censored samples (bees removed at day 7 for potential analysis of gene expression and gut microbiome) were recorded in the dataset and incorporated in the Kaplan–Meier log-rank paired tests to compare survival between treatments. The effects on food consumption between treatments were analyzed by one-way repeated measures ANOVA, while the differences in the dry body weight of bees between treatments were analyzed by one-way ANOVA followed by Tukey’s post hoc tests. An alpha level of 0.05 was used to define the significance for all tests. The data is available in the supplementary materials.

## 3. Results

**Figure 3:**
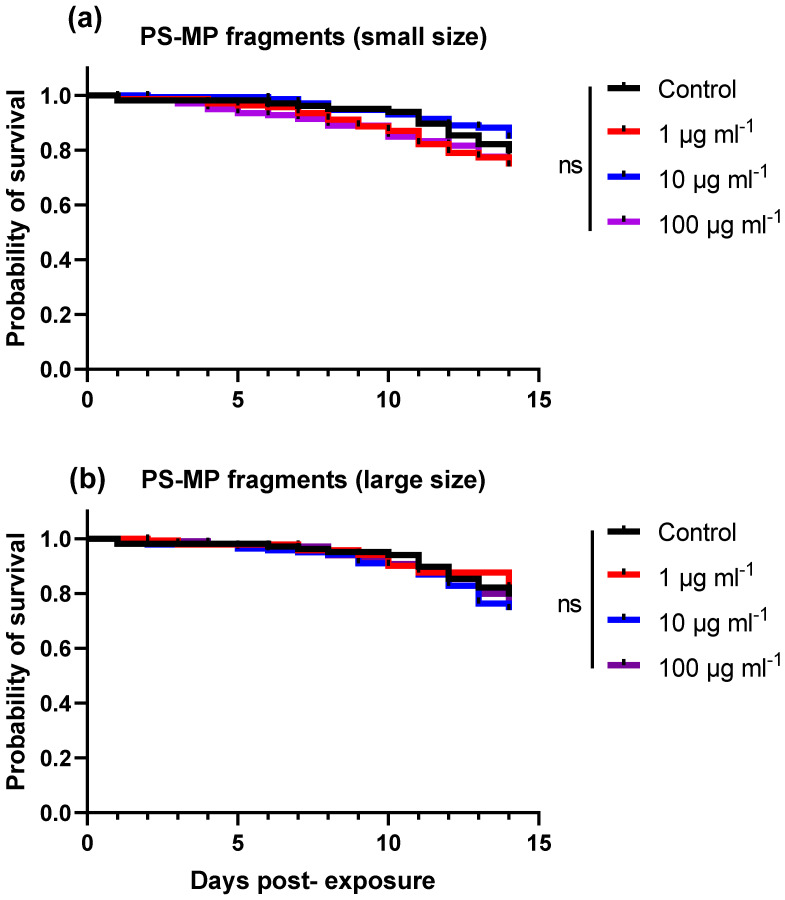
Kaplan–Meier survival curves of honey bees fed sugar syrup containing PS-MP fragments at various concentrations for 14 days. Bees (*n* = 35 bees per cage, *n* = four cages per treatment) were fed ad libitum with various concentrations (1, 10, 100 µg mL^−1^) of either (**a**) small sized (27 ± 17 μm) or (**b**) large sized (93 ± 25 μm) PS-MP fragments. Controls received the same treatments devoid of PS-MP fragments. There were no significant differences between treatments (log-rank (Mantel–Cox), *p* > 0.05).

The survival of the bees was not significantly affected by the concentration of either small- or large-sized PS-MP fragments after 14 days of chronic exposure (log-rank (Mantel–Cox) test: PS-MP fragments-small size, X^2^ = 4.53, df = 3, *p* = 0.20; PS-MP fragments-large size, X^2^ = 2.72, df = 3, *p* = 0.43) compared to the control bees (Figure 3). 

**Figure 4:**
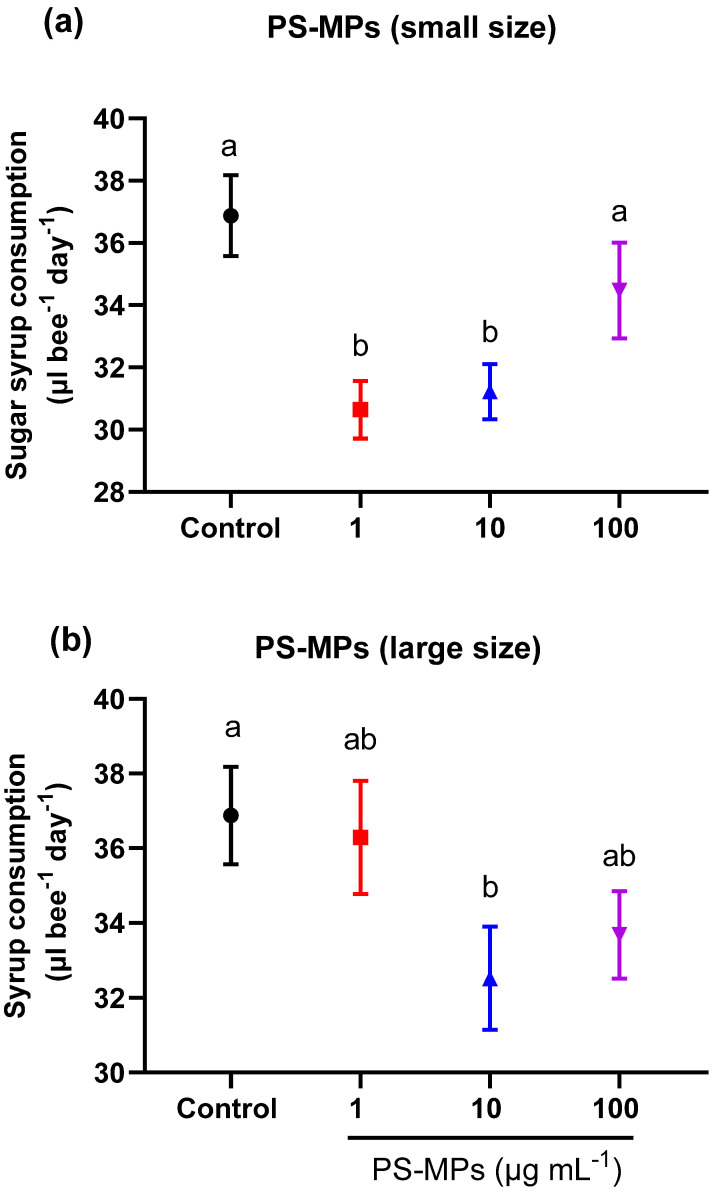
Average sugar syrup consumption (mean ± SEM) of honey bees that were chronically exposed to PS-MP fragments at various concentrations for 14 days. Bees (*n* = 35 bees per cage, *n* = four cages per treatment) were fed ad libitum with various concentrations (1, 10, 100 µg mL^−1^) of either (**a**) small sized (27 ± 17 μm) or (**b**) large sized (93 ± 25 μm) PS-MP fragments. Controls received the same treatments devoid of PS-MP fragments. Different lowercase letters indicate statistically significant differences between treatments (one-way ANOVA with Tukey’s post hoc test, *p* < 0.05).

For small-sized PS-MP treatments, we found a significant reduction in the average daily sugar syrup consumption in the bees fed sugar syrup containing 1 or 10 µg mL^−1^ of small-sized PS-MP fragments compared to the bees fed non-treated sugar syrup (control) (ANOVA, F = 16.76, df = 3, *p* < 0.0001) (Figure 4a). 

For large-sized PS-MP treatments, only the treatment group fed sugar syrup containing 10 µg mL^−1^ of large sized-PS-MP fragments showed a significant reduction in the average daily sugar water consumption compared to the control (ANOVA, F = 5.42, df = 3, *p* = 0.009) (Figure 4b). 

**Figure 5:**
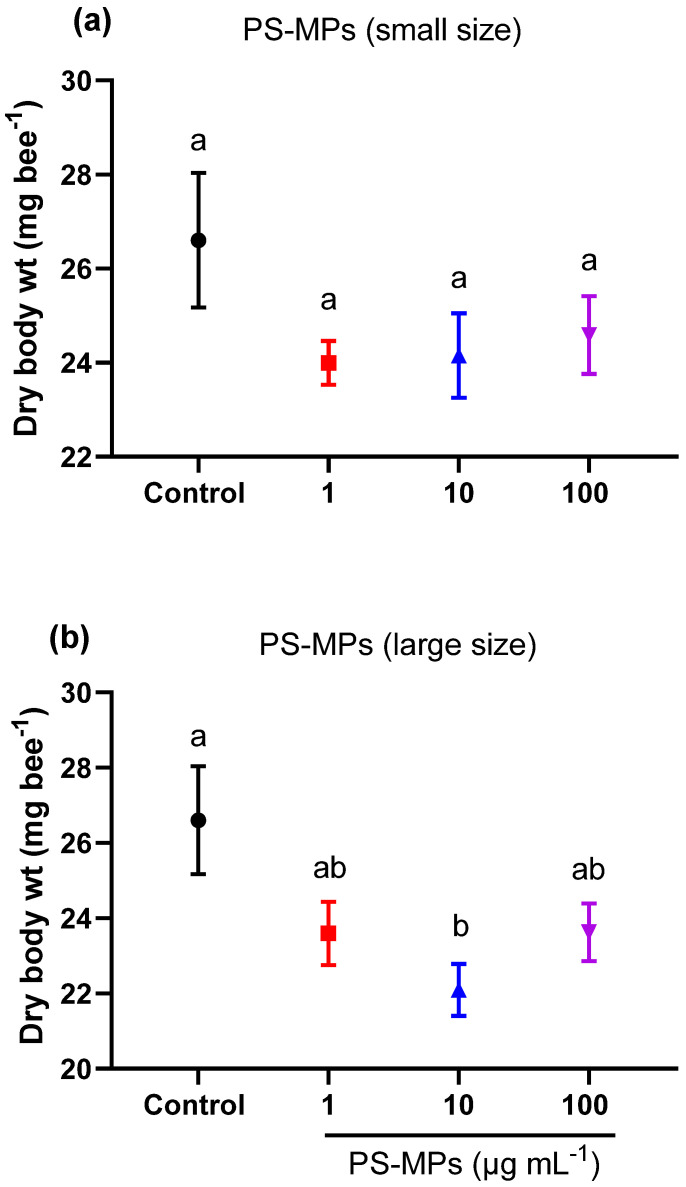
Average dry body weight (±SEM, *n* = 24) of honey bees that were chronically exposed for 14 days to various concentrations (1, 10, 100 µg mL^−1^) of either (**a**) small sized (27 ± 17 μm) or (**b**) large sized (93 ± 25 μm) PS-MP fragments. Controls received the same treatments devoid of PS-MP fragments. Different lowercase letters indicate statistically significant differences between treatments (one-way ANOVA with Tukey’s post hoc test, *p* < 0.05).

We also found a decrease in the body weight in the bees treated with small- or large-sized PS-MP fragments at various concentrations (Figure 5a,b). However, significant differences in body weight compared to the control were only found in bees fed sugar syrup containing 10 µg mL^−1^ of large-PS-MP fragments (ANOVA, F = 3.59, df = 3, *p* = 0.01) (Figure 5b).

## 4. Discussion

A million plastic bottles are purchased every minute around the world, and this number is expected to rise further in the coming years because they are cheap to produce, durable, versatile, and lightweight; their breakdown into PS-MPs could result in an environmental crisis, a global change pressure that poses a global threat to biodiversity and human health [8,38]. The mobility of plastic debris and the lack of information regarding the risks to honey bees have led to an increase in concerns about MP exposure in terrestrial environments [18]. In the present study, we reveal for the first time that chronic exposure to irregularly shaped PS-MP fragments can have an impact on honey bees. 

Microplastic toxicity has been shown to be dependent on MP size, shape, and polymer type [25,39,40,41,42]. The tropical house cricket, for example, experienced a reduction in growth and size at high concentrations of polyethylene terephthalate microfibers in its diet, but no effects were seen when it was fed with polyethylene microbeads [43]. Chronic exposure to PS-MP fragments had no effect on honey bee survival in the current study, as also found when bees were exposed to PS-N/MP in the form of spheres or fibers [22,23,24]. However, when bees were exposed to a mixture of PS spheres of varying sizes and concentrations, a significant decrease in survival was recorded [21] as well as when bees were chronically exposed to polyethylene MP spheres at a higher concentration (50 mg/L) [25]. 

We therefore expected higher mortality after exposure to irregularly shaped PS-MP fragments compared to microbeads, as shown in previous studies [26,41,44]. However, the main effect induced by the PS-MP fragments in our study was a decrease in the bee feeding rate and body weight, which was not observed when the bees were exposed to PS-MP in the form of spheres of fibers [22,24]. This could be explained by the irregular shape and high specific area of PS-MP fragments [41], which could obstruct honey bee mouthparts and the digestive tract (food dilution), and cause other long-term effects such as a false sense of satiation, stomach lining damage, or localized ulcerations, as has been observed in marine animals that consume plastics [45,46].

Although previous research has shown that the toxicity of microplastics may vary with concentration [47,48], significant differences in bee body weight and sugar syrup consumption were only observed at 10 g/mL, not at 100 g/mL of both sizes of PS-MS, in the current study. Even though the cause is unknown, e.g., through disruption of the gut microbiota vs. direct physical impact on the host gut, our results are consistent with previous research that found a significant decrease in the consumption of sugar syrup by honey bees (*A. mellifera*) exposed to PS-MPs spheres at the lowest concentrations (10 mg/L vs. 100 mg/L) [21], (0.5 vs. 5 or 50 mg/L) [22]. Interestingly, the consumption of sugar syrup by another species of honey bee (*Apis cerana*) exposed to PS-MPs spheres at different concentrations (1–10 mg/L) did not change [21]. Additional research is required to determine whether the effects of MPs on pollinators differ depending on concentration and species.

Reduced food intake in bees in response to exposure to PS-MP fragments in the current study led to a decrease in body weight and may, over time, increase mortality, disrupt life history traits, and affect many aspects of reproductive biology, with knock-on effects on the ecological services that honey bees provide [49]. A recent study revealed that honey bees exposed to pristine polystyrene MPs for 15 days lost a significant amount of weight, but only at the nanoscale (100 nm), with no effect on the survival of bees. In addition, PS with a diameter of 100 nm increased the reproduction of *Hafnia alvei* bacteria in guts and may promote the spread of pathogenic bacteria from guts to hemolymph, causing the increased mortality of honey bees [23]. As a result, the observed effects of PS-MP fragments on honey bee food consumption and body weight in the current study could be attributed to a disruption in honey bee gut microbiota, but this hypothesis would need to be validated by gut microbiota analysis. 

A recent field study examined the dynamics and effects of polyester microfiber exposure on honey bee hives and discovered no evidence of chronic toxicity in terms of honey production or bee population size. Bees, on the other hand, can incorporate microfibers from the environment into the hive via their cuticle or through ingestion, transferring them to larvae, honey, and wax [50], with potentially far-reaching negative consequences for the colony’s growth, reproduction, and survival. For example, more research on the health implications of MP persistence in wax is needed to determine whether the MP contamination of wax has any effect on the health of a hive in the long term. Furthermore, the impact of nano-plastics on honey bee health and the risks associated with MPs, their additives, adsorbed contaminants, and pathogens still need to be investigated. 

## 5. Conclusions

This is the first study to investigate the impact of chronic exposure to irregularly shaped PS-MP fragments on honey bee health under controlled conditions. Our findings revealed no effect of PS-MP fragments on honey bee survival, but a decrease in feeding rate and body weight, which may have long-term negative consequences for honey bee fitness.

## Data Availability

The data presented in this study are available in the Supplementary Materials.

## Supplementary Materials

The following supporting information can be downloaded at: https://www.mdpi.com/article/10.3390/toxics11020100/s1, Excel file providing (Sheet 1) units, (Sheet 2) mortality data, (Sheet 3) sugar syrup consumption, and (Sheet 4) body weight.
